# Pilot-Study to Explore Metabolic Signature of Type 2 Diabetes: A Pipeline of Tree-Based Machine Learning and Bioinformatics Techniques for Biomarkers Discovery

**DOI:** 10.3390/nu16101537

**Published:** 2024-05-20

**Authors:** Fatma Hilal Yagin, Fahaid Al-Hashem, Irshad Ahmad, Fuzail Ahmad, Abedalrhman Alkhateeb

**Affiliations:** 1 Department of Biostatistics and Medical Informatics, Faculty of Medicine, Inonu University, Malatya 44280, Turkey; 2 Department of Physiology, College of Medicine, King Khalid University, Abha 61421, Saudi Arabia; 3 Department of Medical Rehabilitation Sciences, College of Applied Medical Sciences, King Khalid University, Abha 61421, Saudi Arabia; 4 Department of Respiratory Care, College of Applied Sciences, Almaarefa University, Diriya, Riyadh 13713, Saudi Arabia; 5 Department of Computer Science, Lakehead University, Thunder Bay, ON P7B 5E1, Canada

**Keywords:** type 2 diabetes, biomarker discovery, metabolomics, machine learning, bioinformatics

## Abstract

Background: This study aims to identify unique metabolomics biomarkers associated with Type 2 Diabetes (T2D) and develop an accurate diagnostics model using tree-based machine learning (ML) algorithms integrated with bioinformatics techniques. Methods: Univariate and multivariate analyses such as fold change, a receiver operating characteristic curve (ROC), and Partial Least-Squares Discriminant Analysis (PLS-DA) were used to identify biomarker metabolites that showed significant concentration in T2D patients. Three tree-based algorithms [eXtreme Gradient Boosting (XGBoost), Light Gradient Boosting Machine (LightGBM), and Adaptive Boosting (AdaBoost)] that demonstrated robustness in high-dimensional data analysis were used to create a diagnostic model for T2D. Results: As a result of the biomarker discovery process validated with three different approaches, Pyruvate, D-Rhamnose, AMP, pipecolate, Tetradecenoic acid, Tetradecanoic acid, Dodecanediothioic acid, Prostaglandin E3/D3 (isobars), ADP and Hexadecenoic acid were determined as potential biomarkers for T2D. Our results showed that the XGBoost model [accuracy = 0.831, F1-score = 0.845, sensitivity = 0.882, specificity = 0.774, positive predictive value (PPV) = 0.811, negative-PV (NPV) = 0.857 and Area under the ROC curve (AUC) = 0.887] had the slight highest performance measures. Conclusions: ML integrated with bioinformatics techniques offers accurate and positive T2D candidate biomarker discovery. The XGBoost model can successfully distinguish T2D based on metabolites.

## 1. Introduction

Type 2 diabetes (T2D) is a chronic disease that represents a global health crisis, with a big concern of rapid escalation worldwide [1]. Due to its multifactorial nature and impaired glucose regulation, T2D significantly burdens both individuals and healthcare systems, leading to an increase in morbidity, mortality, and healthcare costs [1]. Despite extensive research elucidating genetic, lifestyle, and environmental factors related to T2D development, it remains critical to study the molecular mechanisms underlying this disease. Understanding these mechanisms is crucial to developing effective prevention strategies, early diagnostic tools, and therapeutic interventions for T2D [2,3,4,5].

As multi-omics technology advances, Metabolomics, involving the analysis of metabolites in biological specimens [6], will become more important for understanding disease pathogenesis, progression, and therapeutic responses. There have been several studies investigating the metabolic signatures of T2D [6,7]. Machine learning (ML) has been used in recent studies to identify potential biomarkers of T2D progression based on metabolite data; however, as these models lack high accuracy [8], more robust models are needed to identify the role of these substances produced by the body’s metabolism. 

Artificial intelligence (AI) approaches have emerged as a key player in identifying biomarkers for diabetes. Yagin et al. proposed an explainable AI model to predict the outcomes of diabetic retinopathy. The model combining explainable boosting machine (EBM) feature selection and extreme gradient boosting (XGBoost) achieved 91.25% accuracy [9]. In this model, three tree-based ML models are examined, namely, XGBoost, Light Gradient Boosting (LightGBM), and Adaptive Boosting (AdaBoost), to classify metabolites data of T2D patients from normal control patients. Siptroth et al. built a classification model based on microbiome pathway profiles for type 2 diabetes mellitus (T2DM). The model consists of a neural network followed by SHapley Additive exPlanations (SHAP) that identifies the most utilized features in the model to discriminate T2D samples from normal controls. Based on the bacterial pathways data, the model achieved around 84.5% accuracy [10].

This study used comprehensive metabolomics profiling to identify metabolomics biomarkers whose concentrations in serum samples were predicted to distinguish T2D patients from controls. The study used a comprehensive, multi-stage biomarker discovery analysis process to identify metabolomics biomarkers in T2D patients. Fold changes (FCs) analyses, univariate Receiver Operator Characteristic (ROC) analysis, and Partial Least-Squares Discriminant Analysis (PLS-DA) were applied separately to the raw data. Metabolites identified as important across all of these methods were then included as predictors in the machine-learning model for T2D. All these methods were used to validate the biomarker discovery process. The methodology was created to avoid some biomarkers that might incidentally be meaningful in a single method. As a result, the most important biomarker candidates common to all three methods were:

Pyruvate, D-Rhamnose, AMP, pipeclate, Tetradecenoic acid, Tetradecanoic acid, Dodecanediothioic acid, Prostaglandin E3/D3 (isobars), ADP and Hexadecenoic acid. These biomarkers formed the inputs of the frontal diction model. While some studies describe the molecular base interaction of some of the resulting metabolites, including T2D and Pyruvate [11], it is clear from the literature that there is a need for research to examine complete metabolomics profiles. The results showed the individual discriminatory power of each metabolite; Pyruvate came first and achieved the highest discriminatory potential with an area under the curve (AUC) value of 0.832. After comprehensive biomarker discovery, the optimal classification model was established by comparing three tree-based algorithms, which confirmed the accuracy of the candidate biomarker discovery process in T2D patients.

## 2. Materials and Methods

### 2.1. Subjects, and Data

Existing metabolomics data used in this study were publically available from the NIH Common Fund National Metabolomics Data Repository (NMDR) from the Metabolomics Workbench (www.metabolomicsworkbench.org, accessed on 9 December 2023), identified by project ID ST002681. Screening for T2D adhered to the American Diabetes Association Standards of Medical Care guidelines, categorizing patients into control (*n* = 34) or T2D (*n* = 31) groups. Exclusions encompassed pregnant or contraceptive-using women and smokers. Plasma samples underwent mass spectrometry-based metabolomics analysis. Sample size determination, using MetSizeR and setting the false discovery rate to 0.05, indicated a minimum of 14 patients (7 per group). Despite challenges in recruitment, the study surpassed the estimated sample size [12].

### 2.2. Metabolomics Analysis

In obtaining existing metabolomics data, blood samples were collected into fluoride, heparin, and EDTA tubes for glucose, lipid, and metabolomics analyses, respectively. Plasma, obtained after isolation and storage at −80 °C, underwent LC-MS preparation involving resuspension in chilled methanol: acetonitrile: water solution. After centrifugation, the supernatants were subjected to Ultra High-Performance Liquid Chromatography-Mass Spectrometry (UHPLC-MS), a robust analytical technique integrating LC and MS methods [12].

### 2.3. Data Analysis

In demographic and clinical data, qualitative variables were expressed as frequency, while quantitative data were expressed as mean and standard deviation. For comparisons between two groups regarding quantitative data, independent t-test was used and *p* < 0.05 was considered significant. When statistically significant differences were detected, Cohen’s d was calculated for the effect size. Metabolomics data were examined using univariate and multivariate statistical approaches. Data were standardized using the median normalization method, the Pareto scale, and log transformation for multivariate analysis. Significant changes in metabolite levels were assessed using the t-test, and false discovery rates (FDRs) were calculated using the Benjamini-Hochberg approach to reduce false positives [13]. FCs were determined to compare metabolites from T2D patients to controls. Significant results were defined as FDR-adjusted *p* values < 0.05 and FCs ≥ 1.5 (upregulated) or ≤1.5 (downregulated). For exploratory biomarker analysis, we used a Volcano plot to show which metabolites were consistently up or down-regulated in T2D patients compared to healthy controls.

To identify the metabolic signature contributing to group discrimination and to evaluate the predictive performance of potential biomarkers in distinguishing T2D, each metabolite with an AUC value above 70% in univariate ROC was identified. The results are shown with sensitivity, specificity, AUC, and cutoff point for AUC. PLS-DA model VIP plot was another approach utilized to detect biomarkers, and the model’s results were evaluated using accuracy, Q^2^, and R^2^. In addition, the relevance of the PLS-DA model was tested using 1000 permutations. To improve the accuracy and robustness of our analysis, we combined the t-test and FC, as well as the results of ROC analysis and PLS-DA model variable importance, to identify trustworthy biomarker candidate metabolites that contribute significantly to distinguishing T2D patients. To further analyze overlapping metabolites, we used three statistical techniques (FDR-adjusted *p* value < 0.05, AUC > 0.70, and PLS-DA VIP > 1). To differentiate T2D patients, three tree-based ML techniques—XGBoost, LightGBM, and AdaBoost [14,15] were applied. These techniques are popular for ‘omics’-style data analysis since these algorithms have shown to be reliable for high-dimensional data. To validate the performance of ML models and avoid biased results, a 10-fold CV procedure [16] was applied and the results are presented with the mean and 95% confidence interval (CI). To evaluate the prediction performance of the models, accuracy, F1-score, positive predictive value, negative predictive value, sensitivity, specificity, and AUC [17] criteria were calculated.

### 2.4. Classification

In this study, three tree-based classifiers (XGBoost, LightGBM and AdaBoost) were used to determine the T2D class by examining metabolite features. Omics data, such as genomics, transcriptomics, proteomics, and metabolomics, presents unique challenges due to its high dimensionality, complexity, and often small sample size. Gradient boosting algorithms like XGBoost, LightGBM, and AdaBoost offer several advantages in tackling these challenges. XGBoost, LightGBM, and AdaBoost implicitly perform feature selection by assigning importance scores to each feature, allowing identification of the most relevant genes, proteins, or metabolites for the biological process under study. These algorithms incorporate regularization techniques to prevent overfitting, which is particularly critical in high-dimensional omics data where the number of features often exceeds the number of samples. XGBoost, LightGBM, and AdaBoost can capture non-linear relationships between features and outcomes, making them suitable for modeling complex biological processes where interactions and dependencies exist among genes, proteins, and metabolites [14,15].

XGBoost

XGBoost is a gradient boosting model that works by sequentially building decision trees and minimizing a specific objective function at each iteration. XGBoost is represented by Equation (1) as follows:

(1) $${\hat{y}}_{i}=\sum_{k=1}^{K}f_{k}(x_{i})=\sum_{k=1}^{K}w_{k}h_{k}(x_{i})\ldots .$$

where

${\hat{y}}_{i}$ is the predicted output for a sample i; 

K is the number of leaves in each tree;

$h_{k}$ is the prediction of the k-th tree for sample i;

$w_{k}$ are the weights associated with each tree;

$f_{k}(x_{i})$ is the output of the k-th tree for sample i.

AdaBoost

AdaBoost combines multiple weak learners to create a strong learner. The final prediction is a weighted sum of the predictions of all weak learners and can be seen in Equation (2) as follows:

(2) $$F(x)=\sum_{m=1}^{M}f_{m}(x)\ldots .$$

where

F(x) is the final prediction for input x;

M is the number of weak learners;

$\alpha_{m}$ are the weights associated with each weak learner;

$f_{m}(x)$ is the prediction of the m-th weak learner for input x.

LightGBM

LightGBM is based on gradient boosting and can be represented by Equation (3) as follows:

(3) $${\hat{y}}_{i}=\sum_{k=1}^{K}\gamma_{k}h_{k}(x_{i})+\sum_{j=1}^{J}\theta_{j}I(x_{ij}\in \;S_{j})\ldots .$$

where

${\hat{y}}_{i}$ is the predicted output for a sample i; 

K is the number of leaves in each tree;

$h_{k}$ is the prediction of the k-th tree for sample i;

$\gamma_{k}$ are the output values associated with each leaf;

J is the number of bins for each feature;

$x_{ij}$ is the j-th feature of sample i;

$S_{j}$ is the set of bins for the j-th feature;

$\theta_{j}$ are the output values associated with each bin;

$I(x_{ij}\in \;S_{j})$ is an indicator function that evaluates to one if the feature $x_{ij}$ belongs to the set of bins $S_{j}$, and zero otherwise.

## 3. Results

Table 1 presents statistics regarding the demographic and clinical information of the patients (Table 1).

### 3.1. Univariate Statistical Analysis Results

**Figure 1:**
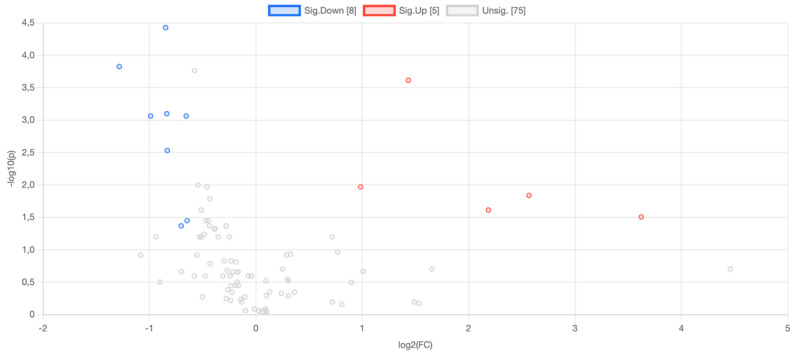
Volcano plot that shows the metabolites fold change (on the x-axis) versus statistical significance (−log10(*p* value)) on the y-axis. Red dots indicate metabolites that are statistically significant and upregulated in T2D compared to control; Blue dots indicate metabolites that are statistically significant and downregulated in T2D compared to control; Colorless dots indicate unsignificant metabolites.

Univariate statistical analyses revealed thirteen biomarker candidate metabolites that differed significantly in T2D patients compared to controls (adjusted *p*-value < 0.05). Our results showed that the metabolite concentration levels of Pyruvate, D-Rhamnose, pipecolate, Tetradecenoic acid, Dodecandioic acid, Prostaglandin E3/D3 (isobars), Hexadecenoic acid and Glycocholate were down-regulated by 0.555, 0.411, 0.560, 0.503, 0.635, 0.561, 0.639 and 0.615 fold, respectively, in T2D patients compared to the control group. In contrast, AMP, ADP, GMP, GDP, and IMP levels in T2D patients were upregulated 2.702, 1.979, 5.927, 4.551, and 12.315-fold, respectively (Table 2, Figure 1).

### 3.2. Univariate ROC Analysis Results

In the initial phase, we scrutinized metabolic processes crucial to the pathophysiology of T2D to enhance our understanding of the metabolic diversity within T2D. Subsequently, we conducted a univariate exploratory analysis of biomarkers to pinpoint more precise metabolomics indicators capable of differentiating T2D from control subjects. To assess the significance of metabolic profiles in the diagnostic screening of T2D patients, we employed univariate ROC analyses to gauge the diagnostic efficacy of metabolites between control subjects and those with T2D. ROC analyses explains the performance of the model on different using different running point in both sensitivity and specificity. In other words, how the classifier performs on both the positive and negative class samples.

We identified eighteen metabolites with an AUC value exceeding 0.7 as potential diagnostic biomarkers for T2D. Individual ROC analyses are presented with cut-off points, AUC values accompanied as well as sensitivity and specificity values, providing insights into the discriminatory potential of selected candidate biomarkers between the two diagnostic groups (T2D/control). Our findings revealed that Pyruvate exhibited the highest discriminatory potential, boasting an AUC value of 0.832, while Octadecanoic acid demonstrated the least discriminatory potential with an AUC value of 0.703 (Table 3).

### 3.3. PLS-DA Model Results

**Figure 2:**
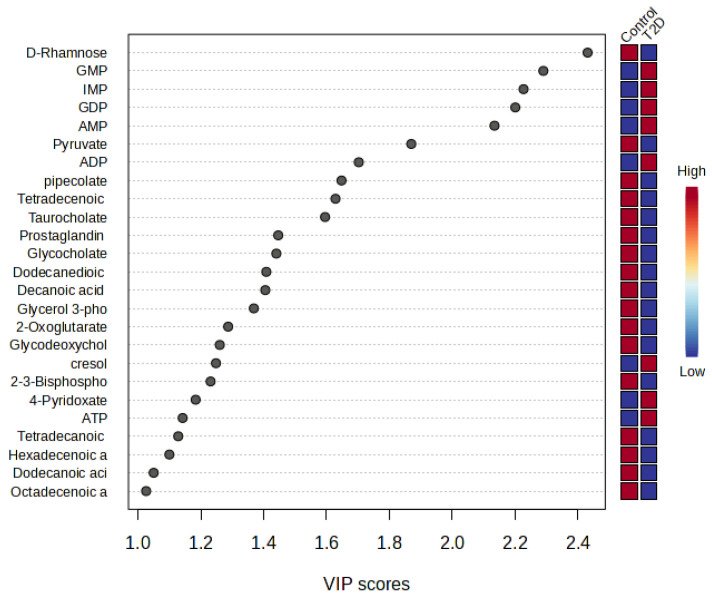
PLS-DA model VIP score graph; Metabolite importance in projection scores of each metabolite and cross-validation results.

The PLS-DA model was applied for metabolomics analyses to differentiate T2D and biomarker discovery. It is an effective evaluator when the features correlated with the class, in addition to the better generalization well to unseen data. The VIP method was used to investigate characteristic volatile metabolites in the PLS-DA model and VIP values > 1.0 indicate calculated group discrimination. In the VIP score plot, D-Rhamnose, GMP, and IMP were the metabolites with the highest scores, and the results showed that these metabolites could play as critical biomarkers to determine discrimination in the PLS-DA model (Figure 2).

**Figure 3:**
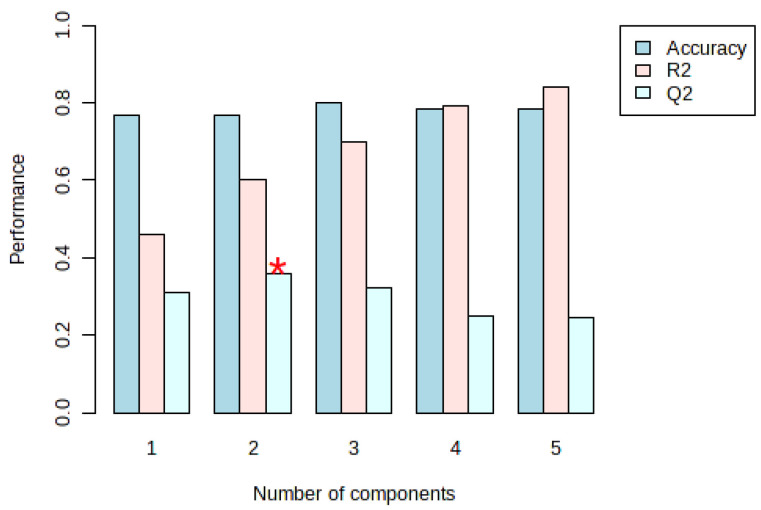
Performance results according to the principal components in the PLS-DA model. Accuracy: In the context of PLS-DA, it can represent the percentage of samples correctly classified by the model. Higher accuracy means a better model fit; R²: In PLS-DA, R² for the model indicates the proportion of variance in the response variable that can be predicted from the predictor variables. An R² value closer to 1 means that the model explains a larger proportion of the variance; Q²: This is a measure of the predictive ability of the model obtained through cross-validation; *: Indicates the component with the maximum Q² value. It shows how well the model can predict new, unseen data. The number of components refers to the number of latent variables used in the PLS-DA model.

**Figure 4:**
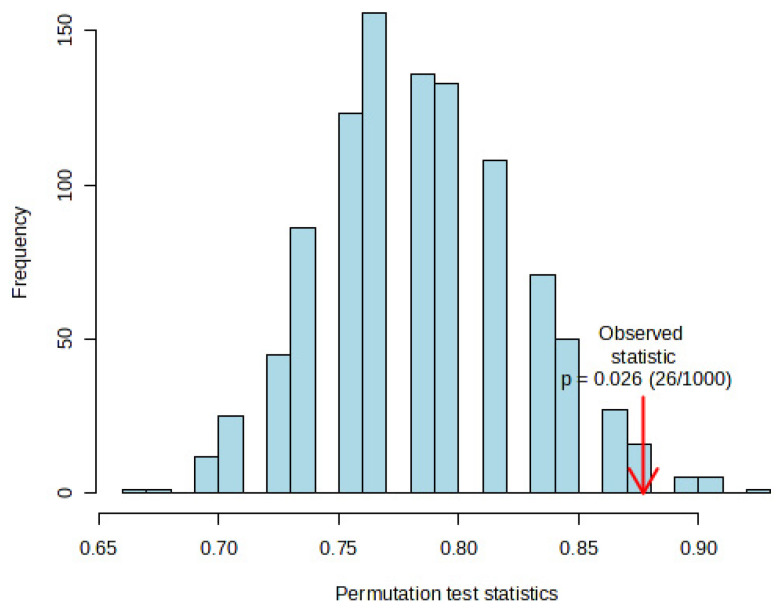
Permutation test results show the frequencies of 1000 permutations with PLS-DA model prediction robustness statistic with *p*-value = 0.026. The y-axis represents the “Frequency” of each statistic observed during the permutation process. The bars show the distribution of the test statistics obtained from permuting the data.

Statistically validated metrics such as accuracy, goodness of fit (R^2^), and goodness of prediction (Q^2^), which evaluate the amount of variation represented by the principal components, were used to examine the performance and robustness of the PLS-DA model. In the PLS-DA model, when the optimal number of components was 5, the accuracy, R^2^, and Q^2^ based on 10-fold cross-validation were 0.785, 0.839, and 0.246, respectively, and the model was robust (Figure 3 and Figure 4, and Table 4).

Pyruvate, D-Rhamnose, AMP, pipecolate, Tetradecenoic acid, Tetradecanoic acid, Dodecanediothioic acid, Prostaglandin E3/D3 (isobars), ADP and Hexadecenoic acid were identified as potential biomarkers for T2D, which were common and validated in FC analysis, univariate ROC analysis and PLS-DA model VIP scores graph results after extensive biomarker discovery analysis.

### 3.4. Diagnostic Model Results

The application of multivariate classification methods to data from metabolomic, genomic, transcriptomic or proteomic technologies is an important area of research [18,19,20,21,22,23]. Currently, evidence on the advantages of one classification method over another is lacking. Therefore, our methodology was to apply three representative tree-based classification methods (XGBoost, LightGBM, and AdaBoost) in parallel to metabolomics data for T2D prediction of interest and compare the results obtained from the different methods. The models were used to classify the results into two groups: T2D patients, and controls.

The performance of the models was evaluated with comprehensive performance metrics and the results were compared. The mean and CI for accuracy, F1-score, sensitivity, specificity, PPV, NPV, and AUC for the developed models are summarized in Table 5, respectively. CV accuracy across the three classification methods ranged from 78.5% to 83.1%. XGBoost model [accuracy= 0.831, F1-score =0.845, sensitivity = 0.882, specificity = 0.774, positive predictive value (PPV) = 0.811, negative-PV (NPV) = 0.857 and AUC = 0.887] had the highest performance measures. Our results showed that the 95% CI for these parameters were 0.740–0.922, 0.757–0.933, 0.725–0.967, 0.589–0.904, 0.648–0.920, 0.673–0.960, and0.828–0.946 respectively (Table 5).

**Figure 5:**
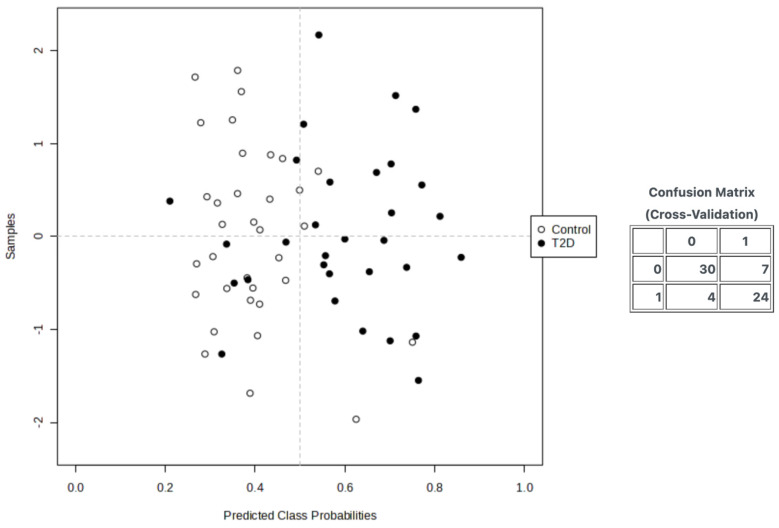
Prediction results and confusion matrix of the class probabilities of the XGBoost model; the x-axis represents the predicted class probabilities for each sample. This probability indicates the model’s confidence in assigning samples to the positive class, which in this context is ‘T2D’. The y axis is labeled ‘Samples’; is an index that vertically separates individual data points for visualization purposes. Data points are categorized into two groups: ‘Control’ (non-T2D) and ‘T2D’. Open circles (‘o’) denote control samples, and filled circles (‘•’) denote T2D samples. The vertical dotted line might represent a threshold for deciding class membership. Samples to the right of this line are more likely to be classified as ‘T2D’, and those to the left as ‘Control’.

The average of the predicted class probabilities of each sample over 10 CVs was determined for the optimal predictive model, and the confusion matrix is summarized in Figure 5. The XGBoost algorithm slightly outperformed the other classifiers with a decent performance over all the evaluation metrics, correctly classifying 24 out of 31 samples with T2D and 30 out of 34 control samples (Figure 5).

## 4. Discussion

T2D is a complex metabolic disorder, and various factors contribute to its development, including genetic, lifestyle, and environmental factors. Although some studies may investigate the potential roles of specific molecules or metabolic pathways in diabetes, the understanding of these relationships is often incomplete [24,25].

In this study, using comprehensive metabolomics profiling, we identified metabolomics candidate biomarkers whose concentrations in serum samples were predicted to discriminate T2D patients from controls. These biomarkers revealed the success of ML-based model in predicting T2D. In the study, statistical and bioinformatics approaches identified metabolic marker in T2D, while the tree-based ML approach, the XGBoost model, was able to successfully discriminate T2D. To improve the interpretability of multivariate prediction models and to more easily characterize T2D patients at the molecular level, extensive biomarker discovery was performed, and overlapping metabolites were identified as biomarkers in FC analysis, univariate ROC analysis, and PLS-DA model VIP scores graph approaches. Statistical tests identified 13 significant metabolites, but multivariate models required only eight metabolites for optimal prediction of T2D. While the identified metabolite characteristics are well supported in the T2D literature, the novel contribution of this study was to evaluate the predictive performance of the biomarker panel in T2D risk prediction. By detecting metabolic changes, the proposed predictive model can support early intervention strategies in T2D and potentially improve patient outcomes. Insights from metabolomics data may inform lifestyle and dietary recommendations targeting high-risk individuals to prevent or delay the onset of T2D.

Pyruvate, D-Rhamnose, AMP, pipecolate, Tetradecenoic acid, Tetradecanoic acid, Dodecanediothioic acid, Prostaglandin E3/D3 (isobars), ADP and Hexadecenoic acid were identified as potential biomarkers for T2D. The XGBoost model slightly outperformed the LightGBM and AdaBoost for predicting T2D using only these eight biomarkers. XGBoost’s optimal predictive model achieved high sensitivity and specificity values in addition to high accuracy and AUC values.

Pyruvate is an important intermediate in glucose metabolism. Thus, impaired insulin signaling in T2D may lead to pyruvate accumulation and potentially contribute to inflammation and other complications [26]. In the presence of oxygen, pyruvate undergoes conversion to acetyl-CoA, subsequently entering the tricarboxylic acid (TCA) cycle for adenosine triphosphate (ATP) generation. Under hypoxic conditions, lactate dehydrogenase facilitates the transformation of pyruvate into lactic acid [27,28]. Research by Lu et al. [29] indicated elevated serum pyruvate concentrations in individuals with T2D, implying heightened glycolytic activity in T2D patients compared to the control group. Similarly, Messana et al. [30] reported significantly increased levels of lactate and citric acid in T2D patients. The TCA cycle, also known as the citric acid cycle, experiences intricate disruptions in T2D. Salek et al. [31] observed elevated blood citrate levels in T2D patients, while Messana et al. [30] demonstrated higher citric acid levels. Intriguingly, the urinary levels of TCA cycle intermediates succinate, fumarate, and malate were notably reduced in T2D patients, necessitating further investigation to elucidate this phenomenon.

AMP (adenosine monophosphate) is involved in energy metabolism and cellular signaling. AMP-activated protein kinases (AMPKs), serving as cellular energy status detectors, are recognized for their significant involvement in the pathophysiology and complications associated with diabetes [32].

Pipecolate is a gut microbial metabolite with potential links to inflammation and insulin resistance. Earlier investigations have established a robust correlation between circulating pipecolate levels and obesity, metabolic syndrome, serving as a predictive indicator for the likelihood of future T2D, hyperglycemia (HG), and increased insulin secretion during early insulin resistance (IR), with a diminished role in the manifestation of advanced IR or T2D [33,34]. Conversely, pipecolate has demonstrated the capacity to enhance insulin secretion in cellular, islet, and animal model systems, suggesting a compensatory mechanism that elevates insulin secretion to preserve glucose homeostasis during early IR. Acting independently on pancreatic β cells, pipecolate regulates insulin secretion in a glucose-dependent manner. Notably, in studies addressing diet-induced obesity, pipecolate acid treatment resulted in significant reductions in body weight, fat accumulation, and fasting glucose levels. The regulation of glycolipid metabolism by pipecolate, irrespective of diet and exercise, suggests its potential as a tool in diabetes management [35]. Our observed decrease in pipecolate levels among T2D patients aligns with prior research, implying that the role of pipecolate in maintaining glucose homeostasis may be overshadowed in established diabetes. Consequently, early pipecolate measurement could emerge as a novel metabolic marker for hyperglycemia, offering predictive value for T2D predisposition and contributing to diabetes risk assessment.

Prostaglandins are lipid signaling molecules with diverse functions. Some prostaglandins may have protective roles in T2D, while others may play a role in inflammation and complications. More research is needed to clarify the roles of specific isoforms in T2D.

In a clinical cohort study, researchers found that prostaglandin levels were an indicator of T2D presence and therapeutic response, independent of known markers of inflammation and obesity disease control. In the current study, prostaglandin was found to be an important biomarker in T2D patients, so our findings provide strong support for future research into prostaglandin as an independent biomarker for long-term disease control in T2D [36].

In the literature, it has been reported that Dodecanedioic acid may act as an intermediate between lipids and carbohydrates and may be used as an alternative fuel substrate in T2D where glucose oxidation is impaired and there is a resistance to glucose storage [37].

In summary, T2D is a multifactorial condition that involves dysregulation of signal transduction, cellular homeostasis, cell apoptosis, dysfunctional adipose tissue, and chronic low-grade inflammation, among others. Identifying early indicators of these abnormalities through biomarkers, such as changes in metabolite patterns, can facilitate intervention and prevention during the prediabetes phase, averting the onset of full-blown T2D. This has spurred increased research into the metabolomics of T2D, leading to the discovery of metabolites linked to obesity, insulin resistance, prediabetes, and T2D across different populations. These potential biomarkers, pending further validation, could complement traditional diabetes markers. Additionally, enzymatic techniques have been developed to create cost-effective tests that enable the clinical validation and usage of these novel biomarkers. As noted by de Castro et al. in 2019 [38], the accessibility of such tests can be enhanced through platforms like point-of-care testing (POCT), which can be utilized in under-served communities or by individuals at home, promoting prevention, early detection, and management of T2D. Implementing effective strategies for diabetes screening, monitoring, and diagnosis is crucial to decrease its prevalence, avert complications, and enhance life quality.

The current study has some methodological limitations. The first limitation is that the metabolites identified as biomarkers are individual molecules and their effects in T2D patients are likely to involve complex interactions and depend on different factors such as dietary habits, lifestyle, and different possible health status. In future studies, studies that combine the effects of different factors and confounding factors with a metabolomic panel are needed. Additionally, this study focused on metabolomic biomarkers, and future studies are needed that will combine different omics levels with patients’ demographic and clinical information regarding age, gender, BMI, various blood values, or medications they use. In conclusion, this study presented a comprehensive biomarker discovery approach based on available metabolomics data from a single center and a tree-based ML model for T2D prediction. Multicenter studies with larger samples are needed to confirm the utility of predictively discovered biomarkers as predictors of complications or treatments observed in T2D patients.

## 5. Conclusions

A comprehensive method of metabolomics data analysis, tree-based machine learning algorithms, and bioinformatics techniques is utilized to identify unique metabolic biomarkers associated with T2D and develop a well-performing predictive model for the condition. The study identified ten potential biomarkers for T2D through rigorous biomarker discovery analyses.

The XGBoost model exhibited a decent performance with an accuracy of 83.1% and an AUC of 88.7%, slightly outperforming LightGBM and AdaBoost classifiers. These findings highlight the potential of ML integrated with bioinformatics for accurate biomarker discovery and disease prediction in T2D.

The identified candidate biomarkers metabolites like pyruvate and pipecolate provide insight into altered glucose metabolism and gut microbial interactions, offering insights into metabolic dysregulation contributing to T2D pathogenesis. This understanding holds promise for enhancing early disease detection and guiding personalized treatment strategies. Further validation studies are warranted to confirm the utility of these biomarkers in clinical practice and their potential translation into therapeutic interventions for T2D management.

## Figures and Tables

**Table 1:** Descriptive statistics on demographic and clinical information.

Variables	Control	T2D	*p* Value (ES)
(male/female)	7/27	8/23	NS
age (years)	50.53 ± 6.5	54.43 ± 8.4 ^a^	0.042 (0.52)
weight (kg)	70.56 ± 11.4	76.63 ± 11.7 ^a^	0.038 (0.53)
BMI (kg/m^2^)	24.04 ± 3.6	27.27 ± 4.6 ^a^	0.002 (0.79)
fasting blood glucose (mg/dL)	96.28 ± 23.3	168.77 ± 75.2 ^a^	<0.001 (1.33)
HbA1C (%)	5.18 ± 0.6	7.24 ± 1.9 ^a^	<0.001 (1.49)
HDL cholesterol (mmol/L)	0.79 ± 0.2	0.65 ± 0.2 ^a^	0.006 (0.70)
LDL cholesterol (mmol/L)	1.27 ± 0.5	1.72 ± 0.6 ^a^	0.002 (0.82)
total cholesterol (mmol/L)	1.60 ± 0.5	2.41 ± 0.6 ^a^	<0.001 (1.47)
triglycerides (mmol/L)	0.92 ± 0.4	1.03 ± 0.6	NS

BMI: body mass index; HbA1C: Hemoglobin A1C; HDL: High-Density Lipoprotein; LDL: Low-Density Lipoprotein; ^a^ Statistically different from control (*p* < 0.05); T2D: type 2 diabetes; ES: effect size; NS: not significant; SD: determines the standard deviation.

**Table 2:** Univariate fold change analysis.

Name	FC	log_2_(FC)	p.ajusted	−log_10_(p)
Pyruvate	0.555	−0.848	<0.001	4.430
D-Rhamnose	0.411	−12.834	<0.001	3.830
AMP	2.702	14.339	<0.001	3.610
Pipecolate	0.560	−0.836	<0.001	3.100
Tetradecenoic acid	0.503	−0.989	<0.001	3.060
Dodecanedioic acid	0.635	−0.654	<0.001	3.060
Prostaglandin E3/D3 (isobars)	0.561	−0.832	0.003	2.530
ADP	1,979	0.984	0.011	1.970
GMP	5.927	25.673	0.014	1.840
GDP	4.551	21.863	0.024	1.610
IMP	12.315	36.224	0.031	1.510
Hexadecenoic acid	0.639	−0.646	0.035	1.450
Glycocholate	0.615	−0.701	0.043	1.370

FC: fold change.

**Table 3:** Univariate ROC analysis.

Name	Cut-Off	AUC	Sensitivity	Specificity
Pyruvate	0.068	0.832	0.806	0.764
D-Rhamnose	0.030	0.826	0.677	0.823
AMP	0.231	0.818	0.677	0.853
Decanoic acid (caprate)	0.093	0.811	0.806	0.676
Tetradecenoic acid	0.136	0.787	0.806	0.705
Pipecolate	0.073	0.776	0.741	0.735
Prostaglandin E3/D3 (isobars)	0.263	0.771	0.870	0.676
Dodecanedioic acid	−0.091	0.761	0.612	0.764
ADP	−0.092	0.759	0.838	0.705
2-Oxoglutarate	−0.050	0.747	0.645	0.823
Phosphoethanolamine	0.12	0.745	0.645	0.764
Octadecenoic acid	0.102	0.744	0.774	0.735
Tetradecanoic acid	−0.061	0.723	0.677	0.705
Dodecanoic acid	−0.046	0.716	0.677	0.764
Hexadecenoic acid	0.060	0.715	0.741	0.735
Octadecadienoic acid	0.079	0.713	0.741	0.647
Hexadecanoic acid	−0.0005	0.706	0.741	0.676
Octadecanoic acid	0.088	0.703	0.806	0.558

AUC: Area under the curve.

**Table 4:** Variance explanation (%) results for each principal component in the PLS-DA model.

Measure	1 Comps	2 Comps	3 Comps	4 Comps	5 Comps
Accuracy	0.766	0.766	0.798	0.783	0.785
R^2^	0.461	0.602	0.699	0.791	0.839
Q^2^	0.311	0.356	0.323	0.250	0.246

**Table 5:** Results of classification performance of tree-based machine learning models in T2D patients.

Metric/Model	XGBoost	LightGBM	AdaBoost
Accuracy	0.831 (0.74–0.922)	0.800 (0.703–0.897)	0.785 (0.685–0.885)
F1-Score	0.845 (0.757–0.933)	0.817 (0.723–0.911)	0.806 (0.709–0.902)
Sensitivity	0.882 (0.725–0.967)	0.853 (0.689–0.95)	0.829 (0.664–0.934)
Specificity	0.774 (0.589–0.904)	0.742 (0.554–0.881)	0.733 (0.541–0.877)
Positive Predictive Value	0.811 (0.648–0.92)	0.784 (0.618–0.902)	0.784 (0.618–0.902)
Negative Predictive Value	0.857 (0.673–0.96)	0.821 (0.631–0.939)	0.786 (0.59–0.917)
AUC	0.887 (0.828–0.946)	0.860 (0.797–0.924)	0.844 (0.781–0.908)

## Data Availability

Data can be requested from the corresponding author with appropriate reason.
